# Unbiased estimation of chloroplast number in mesophyll cells: advantage of a genuine three-dimensional approach

**DOI:** 10.1093/jxb/ert407

**Published:** 2013-12-11

**Authors:** Zuzana Kubínová, Jiří Janáček, Zuzana Lhotáková, Lucie Kubínová, Jana Albrechtová

**Affiliations:** ^1^Charles University in Prague, Faculty of Science, Department of Experimental Plant Biology, Viničná 5, 128 44 Prague 2, Czech Republic; ^2^Institute of Physiology, Academy of Sciences of the Czech Republic, v.v.i., Vídeňská 1083, 142 20 Prague 4, Czech Republic

**Keywords:** Chloroplast counting, confocal microscopy, disector method, mesophyll, coniferous needle structure, Norway spruce (*Picea abies* L. Karst.), profile counting, stereology.

## Abstract

Chloroplast number per cell is a frequently examined quantitative anatomical parameter, often estimated by counting chloroplast profiles in two-dimensional (2D) sections of mesophyll cells. However, a mesophyll cell is a three-dimensional (3D) structure and this has to be taken into account when quantifying its internal structure. We compared 2D and 3D approaches to chloroplast counting from different points of view: (i) in practical measurements of mesophyll cells of Norway spruce needles, (ii) in a 3D model of a mesophyll cell with chloroplasts, and (iii) using a theoretical analysis. We applied, for the first time, the stereological method of an optical disector based on counting chloroplasts in stacks of spruce needle optical cross-sections acquired by confocal laser-scanning microscopy. This estimate was compared with counting chloroplast profiles in 2D sections from the same stacks of sections. Comparing practical measurements of mesophyll cells, calculations performed in a 3D model of a cell with chloroplasts as well as a theoretical analysis showed that the 2D approach yielded biased results, while the underestimation could be up to 10-fold. We proved that the frequently used method for counting chloroplasts in a mesophyll cell by counting their profiles in 2D sections did not give correct results. We concluded that the present disector method can be efficiently used for unbiased estimation of chloroplast number per mesophyll cell. This should be the method of choice, especially in coniferous needles and leaves with mesophyll cells with lignified cell walls where maceration methods are difficult or impossible to use.

## Introduction

Chloroplasts are important organelles of plant photosynthesizing cells as loci where the photosynthetic processes take place. In mesophyll cells, chloroplasts are usually located next to the cytoplasmic membrane adjacent to intercellular spaces to decrease the resistance to CO_2_ diffusion (Terashima *et al.*, 2011). The chloroplast number per cell represents a frequently examined quantitative anatomical parameter, reflecting various leaf internal and external conditions. It can be influenced by internal factors, such as ploidy (Mochizuki and Sueoka, 1955) and environmental factors, e.g. CO_2_ concentration (Wang *et al.*, 2004; Teng *et al.*, 2006). Well-established methods for estimation of the chloroplast number per cell previously used in two-dimensional (2D) space are based on: (i) counting chloroplasts in stomatal guard cells in epidermal peels, (ii) determining the number of chloroplasts in flattened mesophyll cells after maceration, and (iii) counting profiles of chloroplasts in leaf sections.

Chloroplast counting in guard cells of epidermal peels using a light microscope is not problematic, enabling counting of chloroplasts contained in one cell in one focal plane; this has been applied to various plants, e. g. *Beta vulgaris* L. (Mochizuki and Sueoka, 1955), *Solanum tuberosum* L. (Frandsen, 1968), and *Citrullus lanatus* (Thunb.) Matsum. and Nakai. (Sari *et al.*, 1999).

One of the most frequently used methods for estimation of chloroplast number per mesophyll cell in herbaceous plants in 2D is counting chloroplasts in separated mesophyll cells obtained by maceration procedures, as described by Possingham and Saurer (1969). Fixation and maceration of *Spinacia oleracea* L. leaves enabled the separation of mesophyll cells, which were then flattened into a single plane of focus, allowing the chloroplasts to be viewed in a single layer and thus to be easily counted under phase contrast in an optical microscope with an eyepiece graticule. Methods based on these principles were then improved (Possingham and Smith, 1972), and modified protocols have been widely used, e.g. for *Triticum aestivum* L. (Boffey *et al.*, 1979), *Spinacia oleracea* L. (Chaly *et al.*, 1980), *Pisum sativum* L. (Lamppa *et al.*, 1980), *Medicago sativa* L. (Molin *et al.*, 1982), *B. vulgaris* L. (Tymms *et al.*, 1983), and *Chenopodium album* L. (Yamasaki *et al.*, 1996). The maceration process was further adapted and applied to *Arabidopsis thaliana* L. and counting was accomplished after chloroplast thresholding using an image analysis program (Pyke and Leech, 1991; Marrison *et al.*, 1999; Stettler *et al.*, 2009). Sung and Chen (1989) extracted chloroplasts from macerated leaves of *Glycine max* L. Merrill and counted chloroplasts per leaf using a haemocytometer—a special microscope slide with an engraved graticule. To our knowledge, a method for chloroplast counting using maceration has not yet been applied to leaves with thick, lignified cell walls, such as coniferous needles.

Another method frequently used for the estimation of chloroplast number per mesophyll cell in 2D is based on counting chloroplast profiles in semi-thin (1–4 μm thick) physical sections of a leaf using transmission electron and light microscopy (Boffey *et al.*, 1979; Zechmann *et al.*, 2003; Wang *et al.*, 2004; Jin *et al.*, 2011; Simon *et al.*, 2013). This method is frequently used, but its practical application including sampling design is often insufficiently described (Sam *et al.*, 2003; Hayashida *et al.*, 2005; Teng *et al.*, 2006; Gopi *et al.*, 2008). Moreover, in many recent studies, the chloroplast and/or cell profiles in 2D were counted to get an estimation of chloroplast number in 3D, which is a theoretically incorrect approach. For example, the number of chloroplasts per unit of leaf area was estimated from the number of chloroplast profiles in a 0.8 μm thick section observed by light microscopy (Miyazawa and Terashima, 2001; Oguchi *et al.*, 2005) and the number of mesophyll cells per leaf area was determined from the number of cell profiles in 5 μm thick sections (Adachi *et al.*, 2013).

Some further methods have involved analysis of several optical sections from the same specimen, enabling the counting of chloroplasts in 3D directly—either during focusing through the whole thick physical section by a light microscope (e.g. for *T. aestivum* L. and *Triticum monococcum* L.; Ellis and Leech, 1985), focusing through protoplasts in a cell suspension of *Marchantia polymorpha* L. (Bockers *et al.*, 1997), or in a 3D reconstruction created from images of series of optical sections acquired by confocal microscopy. The latter approach was used for determining the chloroplast number per stomatal guard cell in leaves of potato *S. tuberosum* L. (Mozafari *et al.*, 1997), *A. thaliana* L. (Dinkins *et al.*, 2001), *Eucalyptus saligna* Sm. (Xu *et al.*, 2012), and *Glycine dolichocarpa* Tateishi and H. Ohashi (Coate *et al.*, 2012). Chloroplasts in mesophyll cells of *Oryza sativa* L. were counted after comparing photographs from three planes of focus located inside one cell (Hassan and Wazuddin, 2000).

The importance of using a proper method for counting/sampling chloroplasts is obvious, because incorrect approaches can lead to serious bias in the chloroplast number estimation. However, in many studies, the sampling method is not well specified. In general, it is important to note that an unbiased method for sampling/counting particles must sample any of the particles with the same probability (Sterio, 1984). It should be stressed that profiles of particles in a 2D section of a specimen do not represent an unbiased sample of the particles, as the larger particles are sampled (i.e. sectioned) with a higher probability. Moreover, it is known that anatomical parameters often exhibit gradients along the leaf blade (e.g. Pazourek, 1966; Possingham and Saurer, 1969). Thus, a proper design of sampling leaf segments for an analysis is crucial to obtain unbiased results.

For an unbiased estimation of the number of particles in 3D with no assumption about particle shape and size, the optical disector method based on 3D unbiased sampling probe was developed (Sterio, 1984; Gundersen, 1986). This method enables particles of varying shape, such as chloroplasts in a cell and cells within a tissue, to be sampled interactively and counted in an unbiased way. This method has already been used successfully for estimation of mesophyll cell number (Albrechtová and Kubínová, 1991; Kubínová, 1991, 1993, 1994; Kubínová *et al.*, 2002; Albrechtová *et al.* 2007), but to our knowledge it has never been used for chloroplast number estimation.

We hypothesised that the number of chloroplast profiles counted in cell sections in 2D would be different from the number of chloroplasts counted by an unbiased method in 3D. To verify our hypothesis, we compared 2D and 3D approaches to chloroplast counting (i) in practical measurements of mesophyll cells of Norway spruce needles, (ii) in a 3D model of a mesophyll cell with chloroplasts, and (iii) by a theoretical analysis.

## Materials and methods

Norway spruce (*Picea abies* L. Karst.) current-year needles were collected from crowns of 18-year-old trees planted on an experimental site of the Global Change Research Centre, Academy of Sciences, Czech Republic, the Bílý Kříž in Moravskoslezské Beskydy mountains, in 2004 (Urban *et al.*, 2001). Spruce needles were collected from south- and south-west-facing branches from the middle crown part and were stored in a deep freeze until processing (Lhotáková *et al.*, 2008). The images were captured during 2009 and analysed during 2009–2012.

**Fig. 1.**
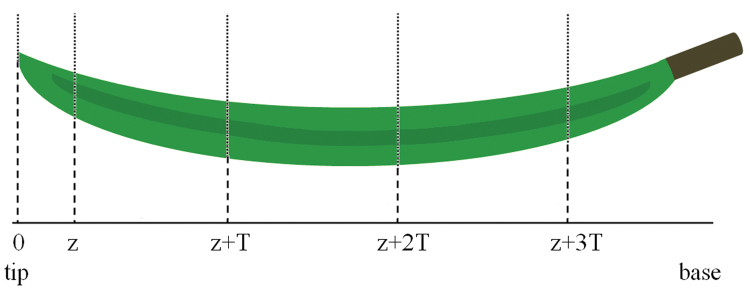
Systematic uniform random (SUR) sampling of positions of cross-sections along the needle. 0, tip; z, position of the first cross-section: a random integer from an interval 0–5 was chosen using a table of random numbers; this number determined the position of the first cross-section (z) in the needle (0 corresponded to 0.5mm from the tip and 1 corresponded to 1mm, up to 5, which corresponded to 3mm from the tip), T=3mm (the interval between subsequent cross-sections). (This figure is available in colour at *JXBV* online.)

Sampling of 0.2mm thick needle cross-sections cut off using a hand microtome and placed into a drop of water was done in a systematic uniform random (SUR) way at intervals of 3mm (Fig. 1).

**Fig. 2.**
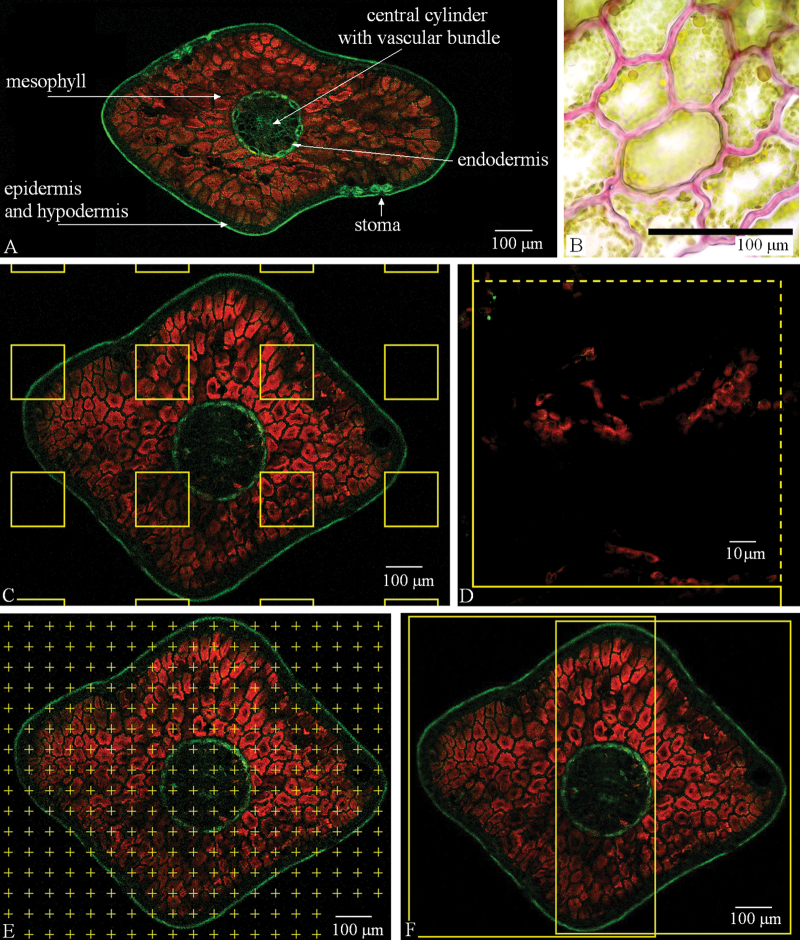
Norway spruce needle cross-section: image acquisition, sampling, and processing. (A) Anatomical structure of a Norway spruce needle in a cross-section. Autofluorescence of chlorophyll in chloroplasts was detected in the red channel and autofluorescence of phenolics was detected in the green channel. (B) Histochemical lignin detection in cell walls (pink) of mesophyll cells using a phloroglucinol/HCl test (O’Brian and McCully, 1981). (C) Sampling frames were superimposed on a needle transverse section using the Rectangles module in Ellipse software. (D) Stacks of optical sections were acquired at the positions of rectangles in (C) with higher resolution. (E) Estimation of needle cross-sectional area by a point counting method using the Point Grid module in Ellipse software (F) Two frames showing the subsequent acquisition of series for counting mesophyll cells. (A, C–F), confocal microscopy: (B) bright-field light microscopy.

The images of the spruce needle cross-sections were captured using a Leica SP2 AOBS confocal laser-scanning microscope (Leica Microsystems, Wetzlar, Germany) with Ar laser excitation of 488nm; phenolics autofluorescence was detected in the green channel (494–577nm) and autofluorescence of chlorophyll in chloroplasts was detected in the red channel (625–710nm) (Fig. 2A). The acquired images were analysed using Ellipse software (ViDiTo, Košice, Slovakia, http://www.ellipse.sk/, last accessed 21 November 2013), which offers special modules for stereological measurements.

**Fig. 3.**
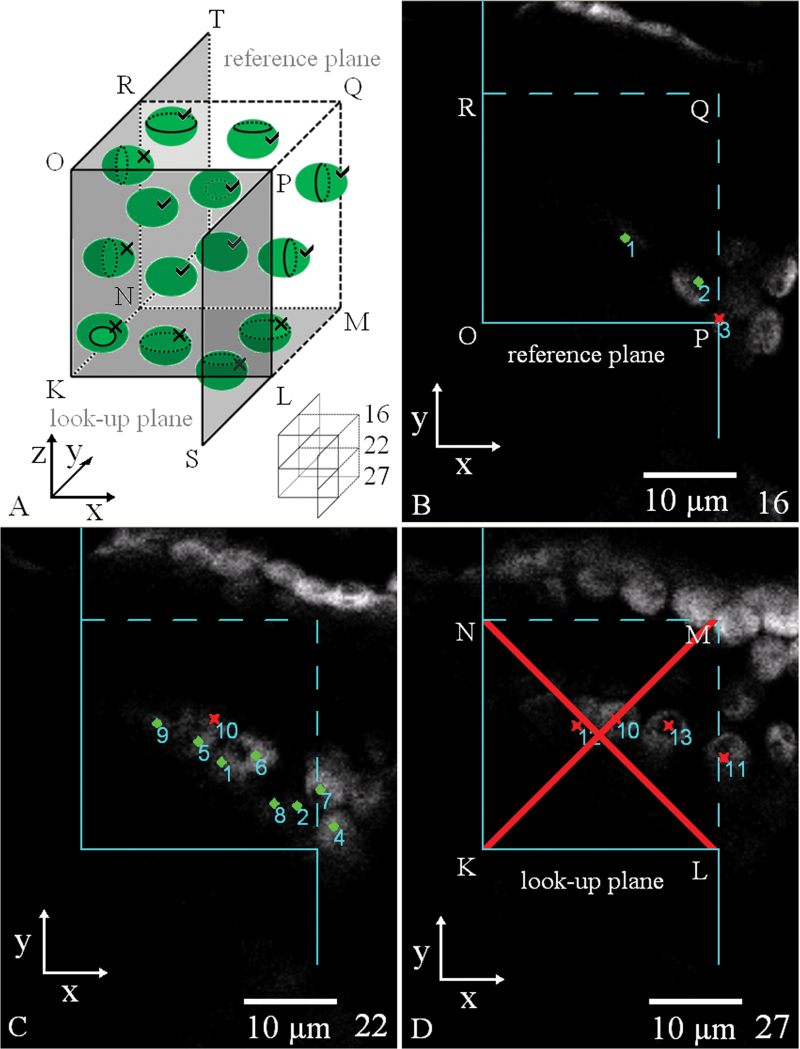
Chloroplast counting using the virtual 3D disector probe. (A) Scheme of the disector probe with chloroplasts. The disector probe is a 3D block. Chloroplasts lying within this block or intersecting its planes are counted, except those intersecting the exclusion planes. The exclusion planes in this scheme are represented by the dark planes and the transparent front plane (all bordered by a full line): the bottom KLMN (so-called look-up plane), KNRO, KLPO, and half-planes PLS and RNT. The top rectangle OPQR is called the reference plane and does not belong to the exclusion planes. Chloroplasts that lie fully inside the probe are always counted. In this scheme, eight chloroplasts are counted (ticked) and six chloroplasts are not counted (crossed). (B–D) Three optical sections of the stack of real serial optical sections acquired by confocal microscopy. Within section numbers 16–27, the disector probe was placed using the Disector module in the Ellipse software, section 16 being the reference plane of the probe (B), section 22 inside the disector probe (C), and section 27 the look-up plane of the disector probe (D). Counted particles are those within the probe not intersecting the exclusion planes: point, counted particle; cross, particle is not counted. In this example, eight chloroplasts were counted (chloroplasts numbers 3 and 10–13 are intersecting the exclusion planes). (This figure is available in colour at *JXB* online.)

**Fig. 4.**
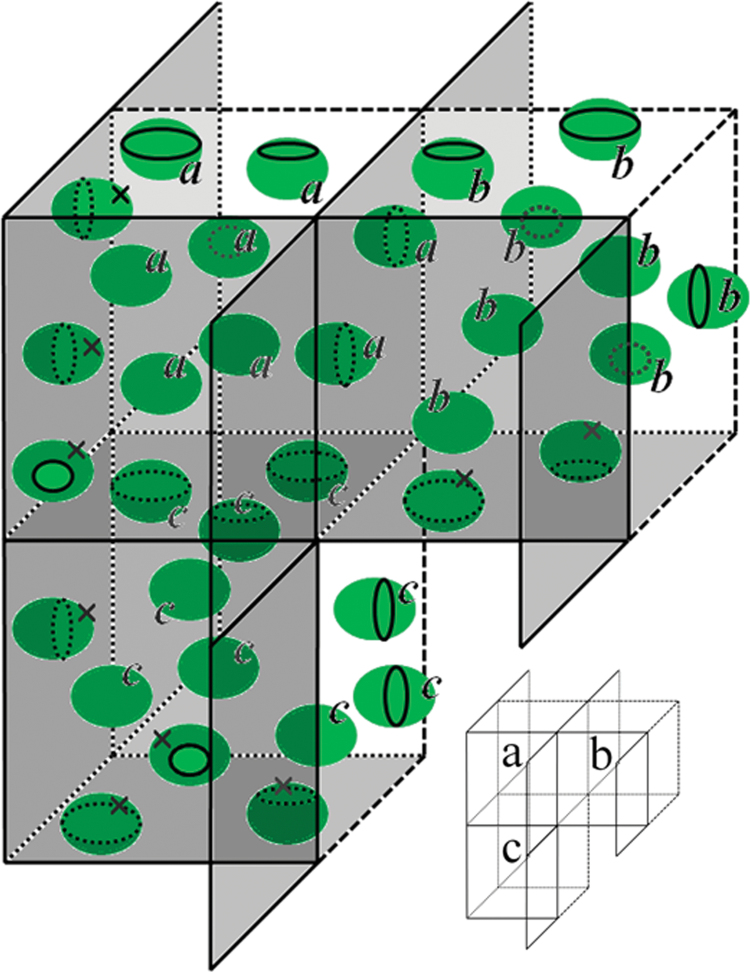
Scheme of the hypothetic spatial mosaic of 3D disector sampling probes illustrating that particles are unambiguously sampled by only one disector sampling probe. The probes a, b, and c are located next to each other. The exclusion planes in this scheme are represented by grey planes. Particles are labelled according to the label of the probe they are counted in. In this scheme, in probe *a* eight chloroplasts are counted (labelled *a*), in probe *b* eight chloroplasts are counted (labelled *b*), and in probe *c* nine chloroplasts are counted (labelled *c*). Chloroplasts labelled by crosses are not counted by any of the three probes shown here, but they would be unambiguously sampled by adjacent hypothetical probes. (This figure is available in colour at *JXB* online.)

The Ellipse Disector module (Tomori *et al.*, 2001) enables application of a virtual 3D probe to the stack of serial optical sections. It is possible to browse through the successive optical sections and to mark the selected particles (Figs. 3B–D). The optical disector probe (Sterio, 1984, Gundersen, 1986) represents a virtual 3D block with three exclusion planes (Fig. 3A). The bottom look-up plane belongs to exclusion planes together with two side planes. The reference plane on the top does not belong to the exclusion planes. Particles lying within this block or intersecting its planes, except the exclusion ones, are counted (Fig. 3). The particles are sampled without bias if each particle has the same probability of being sampled, i.e. if the 3D space is filled in by the spatial mosaic of the shifted copies of the disector probe and each particle in the space is sampled by one and only one shifted copy of the 3D sampling probe (Fig. 4).

At first, the image of an entire cross-section was captured using a dry plan apochromatic 10× objective [numeric aperture (NA) 0.4] with resolution 1024×1024 pixels (1500×1500 µm). From these images, the areas of needle cross-sections and the proportions of mesophyll were estimated by the point counting method (Weibel, 1979) using the Ellipse Point Grid software module (Fig. 2E). The spruce needle volume was estimated by Cavalieri principle (Gundersen and Jensen, 1987): the mean of the areas of cross-sections was multiplied by the length of the needle.

For mesophyll cell counting, 30 µm thick stacks comprising 16 serial optical sections 2 µm apart were acquired by using a plan apochromatic 20× water-immersion objective (NA 0.7) with resolution 2048×2048 pixels (750×750 µm) (Fig. 2F, Supplementary Videos S1 and S2, at *JXB* online). Several stacks were acquired in each cross-section to cover its whole area; altogether, 157 stacks were acquired. The height of the disector probe was 10 μm, placed in the middle of captured stacks, i.e. within a substack of six optical sections 2 μm apart, the size of the sampling frame was adjusted to cover the whole cross-section area. A double disector (Gundersen, 1986) was used: the probe was used in both directions, i.e. at first, cells were counted by the disector with the reference plane and look-up planes in the positions shown in Fig. 3A, and then additional cells were counted by the disector with the reference plane set to the position of the former look-up plane and vice versa. Thus, the total height of the probe was twice its real height (20 μm, in this case). The needle volume in the stack was determined by multiplying the height of the double disector and the needle area estimated from the cross-section captured by a 10× objective. The mesophyll cell density (number of cells per needle volume) was estimated by the formula (Sterio, 1984; Gundersen, 1986):


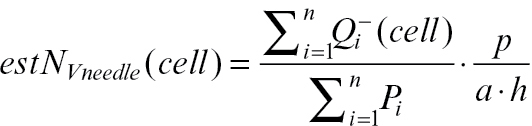


where *estN*_*Vneedle*_ *(cell)* is the estimated number of mesophyll cells per needle volume, 
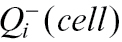
 is the sum of all sampled cells in all disector probes within a needle, *∑P*_*i*_ is the sum of all points falling within a needle in all disector probes (for calculation needle volume in each probe) used for cell counting, *p* is the number of test points in a grid in a sampling frame used for cell counting, *a* is the area of the disector sampling frame (the base plane of the 3D probe) used for cell counting, and *h* is the height of the disector probe used for cell counting

For chloroplast counting, the positions of captured stacks were selected by random application of rectangle grid in the Rectangles module of the Ellipse software (ViDiTo, Košice, SR) (Fig. 2C) on the images captured by a 10× objective, while the size of the rectangles was set equal to the size of the field of view and the distance between the rectangles was set to capture on average 24 stacks of optical sections per spruce needle. In each position, 20 µm thick stacks comprising 41 serial optical sections 0.5 µm apart were acquired using a plan apochromatic 63× water-immersion objective (NA 1.2) with zoom 2× and resolution 512×512 pixels (119×119 µm) (Fig. 2D). Altogether, 464 stacks were acquired and used for counting chloroplasts by applying the disector method.

The dimensions of the disector sampling frame for chloroplast counting in the middle of the stack were set to 500×500 pixels (116×116 μm) and the height of the probe was 5.5 μm, as there were 12 optical sections included, 0.5 μm apart. To estimate the volume of the spruce needle within each disector probe, the needle area in the middle image of the stack was determined by the point-grid method and multiplied by the disector height. The chloroplast density (number of chloroplasts per needle volume) was estimated by the formula (Sterio, 1984; Gundersen, 1986):


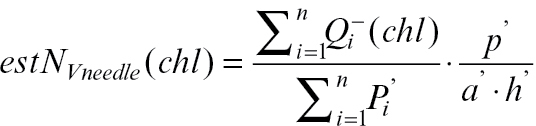


where *estN*_*Vneedle*_ *(chl)* is the estimated number of chloroplasts per needle volume, 
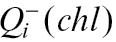
 is the sum of all sampled chloroplasts in all disector probes within a needle, *∑P’*_*i*_ is the sum of all points falling within a needle in all 3D probes (for calculation of needle volume in each probe) used for chloroplast counting, *p’* is the number of test points in a grid in a sampling frame used for chloroplast counting, *a’* is the area of the disector sampling frame (the base plane of the 3D probe) used for chloroplast counting, and *h’* is the height of the disector probe used for chloroplast counting.

The estimated number of chloroplasts per mesophyll cell was calculated by the ratio of the chloroplast number and the mesophyll cell number per needle volume:


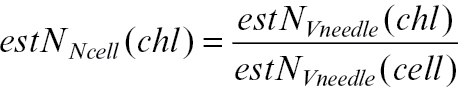


where *estN*_*Ncell*_ *(chl)* is the estimated number of chloroplasts per mesophyll cell, *estN*_*Vneedle*_ *(cell)* is the estimated number of mesophyll cells per needle volume, and *estN*_*Vneedle*_ *(chl)* is the estimated number of chloroplasts per needle volume.

The profiles of chloroplasts and cells in 2D images were counted in the same stacks as those used for 3D measurements in the middle optical sections using the 2D unbiased sampling frame (Gundersen, 1977) (Fig. 2D). The number of chloroplasts per cell was calculated as the ratio of the chloroplast profile number per needle area to the cell profile number per needle area, while the needle area was estimated previously by the point counting method (Weibel, 1979).

In order to obtain comparable results by 2D and 3D methods, we calculated the chloroplast number per cell in each needle cross-section (68 in total) by both methods. In the 3D method, we summed the number of chloroplasts per volume as estimated by the disector method in each cross-section and divided it by the sum of the estimated number of cells in each cross-section. In the 2D profile counting method, we summed the estimated number of chloroplasts per area in each cross-section and divided it by the sum of the estimated number of cells in each cross-section. Acquired values were compared using statistical analysis (paired *t*-test) in NCSS 2000 program (Number Cruncher Statistical Systems, Kaysville, Utah, USA).

**Fig. 5.**
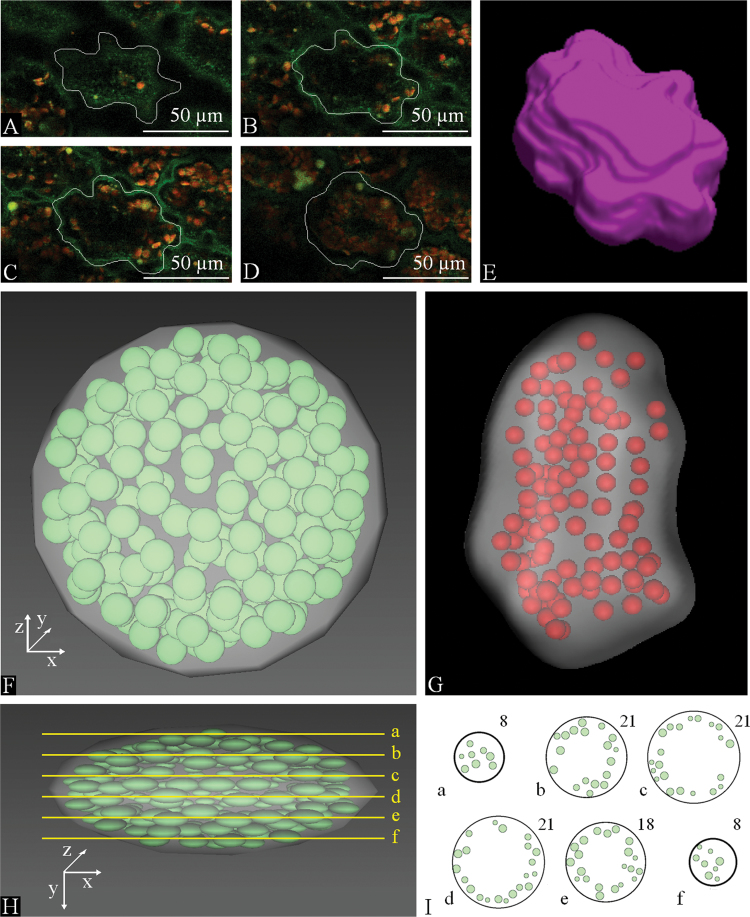
3D reconstruction and 3D model of a Norway spruce mesophyll cell. (A–D) Different optical sections of a mesophyll cell in a stack of images captured by a confocal microscope by using an objective 20×. (E) 3D reconstruction of the surface of the mesophyll cell from the confocal image stack shown in (A)–(D) using Ellipse software. (F) Face view of a model of a mesophyll cell with 210 chloroplasts made in Cortona 3D software. (G) 3D reconstruction of the mesophyll cell with chloroplasts from a stack of optical sections from confocal microscopy using Ellipse software. The positions of chloroplasts do not correspond to the *in vivo* state. (H) Side view of the model from (F) with six lines indicating planes of cross-sections. (I) 2D sections through the model cell shown in (F) and (H). Letters indicate the position of section planes in (H). Numbers indicate the number of chloroplast profiles in each of these sections.

For visualization of mesophyll cell surface (Fig. 5A–E) and 3D chloroplast arrangement in a mesophyll cell (Fig. 5G), the 3D reconstructions were created based on processing series of optical sections 2 µm apart acquired by a confocal microscope using a 20× objective. A model of a simplified mesophyll cell with 210 chloroplasts was made in the Cortona software (Fig. 5F, H, Supplementary Video S3 at *JXB* online) in order to demonstrate comparison of the 3D disector and the 2D profile counting methods.

For maceration of needle mesophyll samples, in order to count chloroplasts directly in separated cells, different methods were tested: a 1M aqueous solution of HCl (Possingham and Saurer, 1969); a 3.5% aqueous solution of glutaraldehyde and 0.1M aqueous solution of Na_2_EDTA (Boffey *et al.*, 1979); and a solution of 10% aqueous CrO_3_ and 10% aqueous HNO_3_ (O’Brian and McCully, 1981). A phloroglucinol/HCl test (O’Brian and McCully, 1981) was applied to demonstrate lignification of mesophyll cell walls (Fig. 2B).

## Results

The Norway spruce needle structure (Fig. 2A) is composed of a one-cell-layer epidermis with highly cutinized periclinal cell walls on the needle surface; below the epidermis, there is one-cell layer of sclerenchymatic hypodermis not present below the stomata, followed by mesophyll and a central cylinder with a vascular bundle surrounded by transfusion tissue. Mesophyll cells contain chloroplasts and have an irregular shape with lobed anticlinal cell walls and are prolonged in the direction perpendicular to the needle surface (Fig. 5E), forming layers of tightly connected mesophyll cells surrounded by intercellular spaces (Supplementary Videos S1 and S2, Supplementary Fig. S1 at *JXB* online).

Numerous maceration methods were tested in order to count chloroplasts directly in separated spruce mesophyll cells. However, the spruce mesophyll cells kept connected together by the middle lamella and cell walls. We concluded that maceration methods were unsuccessful due to lignification of mesophyll cell walls detected histochemically (Fig. 2B) and observed regularly in our previous study (Soukupová *et al.*, 2000).

**Fig. 6.**
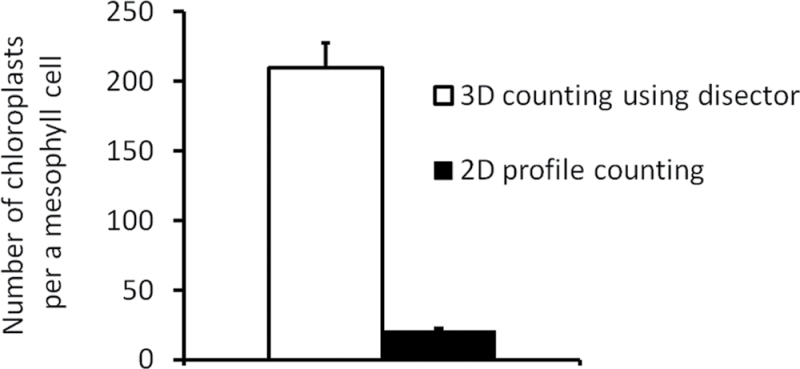
Comparison of 3D- and 2D-based methods for chloroplast number estimation. The mean number of chloroplasts per mesophyll cell in Norway spruce needle estimated by 3D disector was 209.65±17.44 (mean±SE) (open column), and by profile counting in 2D (filled column) was 20.92±1.32. This result was statistically significantly different (*P*<0.001).

The average Norway spruce needle volume was 8.17±1.00mm^3^ ([mean ± standard error (SE)] and the proportion of mesophyll in the needle volume was 72.0±0.8 %. The average number of chloroplasts per mesophyll cell estimated by profile counting in 2D was 20.92±1.32, while the average number of chloroplasts estimated by the disector probe in 3D was 10 times higher, i.e. 209.65±17.44 (Fig. 6). Unsurprisingly, statistical analysis showed a significant difference between results obtained in 2D and 3D. The methods of chloroplast counting in 2D and 3D thus yielded estimates of the chloroplast number per mesophyll cell that were different by one order of magnitude.

To visualize differences in the results yielded by both methods, we used the 3D model of a mesophyll cell (Fig. 5F, H, I, Supplementary Video S3). We made 111 sections through the cell model and counted profiles in each section. The mean number of particle profiles per section was 17.23±0.69 (mean±SE). However, there were 210 particles in the model cell. Our test showed that the average number of chloroplasts estimated by profile counting was more than 10 times underestimated in comparison with the real number of chloroplasts in the model. This is clearly demonstrated in Fig. 6.

The difference between both estimators tested can be also explained theoretically as shown below. The number of chloroplasts per mesophyll cell can be calculated by:


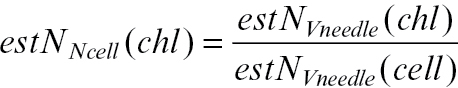


Instead of direct counting of *N*, it could be estimated by estimations of densities 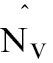:


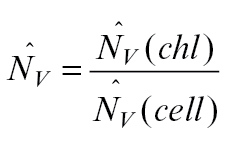


obtained by stereological methods. 
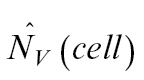
 should be estimated with a small error, 
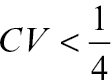
, because it is not possible to divide by zero, and 
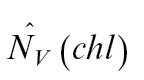
with an error of about 
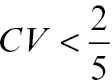
 (Marsaglia, 2006). The number of chloroplasts cannot be directly estimated by counting profiles in the section, because the areal density of profiles is related not only to density *N*_v_ but also to height *h*: 
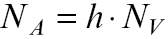
. If we count the ratio of estimations,


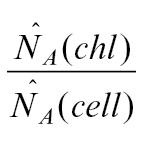


we get close to the value


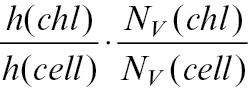


which is different from the desired value by the ratio of the height of chloroplasts to the height of mesophyll cells:


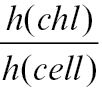


The chloroplasts are much smaller than mesophyll cells (chloroplast mean height is approximately 4 μm, while the cell mean height is approximately 40–60 μm); therefore, the theoretically expected difference is high, also of one order of magnitude.

## Discussion

Counting chloroplast profiles in 2D yielded values of chloroplast number per cell that were 10 times underestimated in comparison with the mean number of chloroplasts estimated by the unbiased disector method in 3D. We determined that the systematic shift in an estimated value is inversely related to the ratio of the height of chloroplasts to the height of mesophyll cells. The 3D model also showed that neglecting the 3D appearance of the cell led to the underestimation of the number of chloroplasts (Fig. 6). All our practical and theoretical tests thus clearly showed that the frequently used method for chloroplast number estimation by counting profiles of particles from 2D sections (e. g. Boffey *et al.*, 1979; Sam *et al.*, 2003; Wang *et al.*, 2004; Hayashida *et al.*, 2005; Teng *et al.*, 2006; Gopi *et al.*, 2008) yielded biased estimates and that the results may be one order of magnitude different from the real chloroplast numbers.

A review of the previously reported results on the number of chloroplasts per mesophyll cell with focus on the method used is presented in Supplementary Table S1 at *JXB* online. The studies were conducted on various plant species, considering herbaceous species in the majority of cases. For example, the extreme variability can be seen in the number of chloroplasts in *Arabidopsis* mesophyll cells estimated by various methods. The number of chloroplasts varied from eight to 10 chloroplasts per cell, based on counting chloroplast profiles in thin sections (Teng *et al.*, 2006; Jin *et al.*, 2011), to more than 100 chloroplasts per cell if the number was determined by the maceration method (Pyke and Leech, 1992; Marrison *et al.*, 1999), or even more than 200 (Meyer *et al.*, 2006).

It is generally known that the number of chloroplasts depends on plant internal factors—developmental stage, age, and species genotype (reviewed by Possingham, 1980)—or environmental factors, although the methodical bias could conceal effects of these factors. Authors often do not describe the sampling design and the method used for chloroplast counting. Nevertheless, sampling design is crucial for yielding unbiased results, as anatomical gradients within a leaf blade were demonstrated also for the number of chloroplasts per cell (Dean and Leech, 1982). Gradients in leaf anatomical parameters, e.g. the number of chloroplasts per cell, also exist in dependence on distance of a leaf from the root system (Possingham and Saurer, 1969).

The chloroplast number per cell in conifers was estimated by profile counting to be six to 16 chloroplasts per cell in different species sampled in August (Maslova *et al.*, 2009) (Supplementary Table S1). These numbers seem to be very low and may not reflect the real chloroplast number in a mesophyll cell. For example, the number of chloroplasts per pair of stomatal guard cells in epidermal peels varies between 10 and 20 (Mochizuki and Sueoka, 1955; Frandsen, 1968; Nicholson, 1981; Qin and Rotino, 1995; Sari *et al.*, 1999) and the mesophyll cells are obviously larger than guard cells. In flat cells, such as the stomatal guard cells, it is possible to observe the chloroplasts practically in one optical section and to count them correctly in contrary to the mesophyll cells, which are larger and thicker, so it is unlikely that profiles of all of the chloroplasts within a cell would be present in a single optical section.

Chloroplast counting in separated mesophyll cells after leaf maceration (e.g. Possingham and Saurer, 1969; Stettler *et al.*, 2009) is appropriate in the case when the chloroplasts in the specimen are not overlapping. Counting of chloroplasts in a solution from the macerated leaf segments (Sung and Chen, 1989) does not enable the determination of chloroplast number per cell as the number of mesophyll cells, from which the chloroplasts present in a solution are released, is not known. Applying the maceration method to coniferous needles is problematic or even impossible, as shown in our study, as they contain phenolic compounds and lignin in cell walls (Fig. 2B), as proved in a histochemical study by Soukupová *et al*. (2000).

Counting chloroplasts in cells directly during focusing through the specimen using conventional light microscopy (Ellis and Leech, 1985; Bockers *et al.*, 1997) can be applied if entire cells can be focused through and the chloroplasts are sparsely distributed in cells; however, chloroplasts usually tend to be densely packed along the cytoplasmic membrane. Determining the chloroplast number per cell in 3D reconstructions made from a series of confocal microscope images (Mozafari *et al.*, 1997; Dinkins *et al.*, 2001; Coate *et al.*, 2012; Xu *et al.*, 2012) can yield an unbiased estimate if SUR sampling is applied and a sufficient number of cells is analysed. However, this is a much more time-consuming approach than application of the disector method.

In order to get unbiased results, it is also important to apply unbiased sampling of locations within the leaf where the measurement is performed. Many authors of previous studies reporting the chloroplast number per mesophyll cell do not explain the sampling design in a sufficient detail. However, a SUR sampling, along with both assumptions—the unbiased method for counting particles and the sufficient number of measurements—are essential for getting unbiased and accurate results (Sterio, 1984).

In the present study, sample preparation and image acquisition were carefully designed according to the principle of SUR sampling (Gundersen and Jensen, 1987). Needles collected from Norway spruce trees were stored frozen before processing; freezing of needles does not influence various geometrical parameters of mesophyll (Lhotáková *et al.*, 2008). Similarly, the number of mesophyll cells and the number of chloroplasts cannot change during the needle storage. Norway spruce chloroplasts kept their chlorophyll fluorescence activity, even after 5 years of storage in a freezer, and were perfectly suitable for confocal microscopy. To eliminate the possible shrinkage of a tissue caused by cutting, the dry stem pith of *Sambucus nigra* L. was used to fix the sample during cutting. It was found that some cells might have been pulled out of the specimen during cutting (Lhotáková *et al.*, 2008), and this is why the disector probe was placed in the middle of a stack.

In general, plant mesophyll cells contain a large central vacuole and, *in vivo*, chloroplasts are usually located in the peripheral cytoplasm, close to the cell wall. However, the spatial distribution of organelles may be disrupted by freezing. Therefore, we are aware that our 3D models of mesophyll cells (Fig. 5) do not correspond to *in vivo* chloroplast arrangement; however, they illustrate well the size of Norway spruce mesophyll cells, their variable and irregular shape, as well as the number and size of chloroplasts within a cell. The particular mesophyll cell chosen for 3D reconstruction was a smaller one, so that it was possible to focus through the entire cell. Most mesophyll cells were too high to be focused through; therefore, a method based on direct counting of chloroplasts within the entire cell could not be used. The present 3D model of mesophyll cell (Fig. 5F, H, I) was constructed such that it contained the mean number of chloroplasts obtained in our study.

## Conclusions

Based on the above-presented lines of evidence, we conclude that the presented optical disector method for chloroplast counting, using stacks of confocal microscopic images acquired from thick tissue sections accompanied by SUR sampling, is a very efficient method for estimating chloroplast number per mesophyll cell. While using the optical disector method for chloroplast counting, an important consideration that a mesophyll cell is a 3D structure is taken into account. We propose this method as a universal unbiased one. It should be the method of choice, especially in conifers or other xeromorphic leaves with mesophyll cells with lignified walls, where maceration methods are difficult or impossible to apply.

## Supplementary data

Supplementary data are available at JXB online.

Supplementary Figure S1. Norway spruce needle longitudinal median section by confocal microscopy using 20× objective.

Supplementary Table S1. Review of studies with results on the number of chloroplasts per mesophyll cell of different plant species (most studied plant species: families *Brassicaceae*, *Fabaceae*, *Chenopodiaceae*, and *Poaceae*, and other families; coniferous species) with focus on the method used.

Supplementary Video S1. 3D stack of 16 serial optical cross-sections 2 µm apart acquired by confocal microscopy using a 20× objective.

Supplementary Video S2. 3D reconstruction of the mesophyll arrangement created by volume rendering from images of 16 serial optical sections rotating in 3D space.

Supplementary Video S3. 3D model of a simplified mesophyll cell with 210 chloroplasts (modelled by surfaces of oblate ellipsoids) made in IRIS Explorer (NAG, UK).

Supplementary Data

## Abbreviations:

2D: two-dimensional
3D: three-dimensional
SUR: systematic uniform random.
