# Supplementary material for: Unbiased estimation of chloroplast number in mesophyll cells: advantage of a genuine three-dimensional approach
Source: J Exp Bot. 2013 Dec 11;65(2):609–20. doi: 10.1093/jxb/ert407 (PMC3904715; doi:10.1093/jxb/ert407)
Supplement: Supplementary Data [file supp_65_2_609__index.html]

Unbiased estimation of chloroplast number in mesophyll cells: advantage of a genuine three-dimensional approach — Unbiased estimation of chloroplast number in mesophyll cells: advantage of a genuine three-dimensional approach — Supplementary Data 

# Unbiased estimation of chloroplast number in mesophyll cells: advantage of a genuine three-dimensional approach

## Supplementary Data

Data files

**Files in this Data Supplement:**

- Supplementary Data - Supplementary Data
- Supplementary Data - Supplementary Data
- Supplementary Data - Supplementary Data
- Supplementary Data - Supplementary Data
