# Supplementary material for: Unbiased estimation of chloroplast number in mesophyll cells: advantage of a genuine three-dimensional approach
Source: J Exp Bot. 2013 Dec 11;65(2):609–20. doi: 10.1093/jxb/ert407 (PMC3904715; doi:10.1093/jxb/ert407)
Supplement: Supplementary Data [file supp_ert407_jexbot105429_file004.pdf]

Journal of Experimental Botany

Unbiased estimation of chloroplast number in mesophyll cells: Advantage of genuine 3D approach

Zuzana Kubínová, Jiří Janáček, Zuzana Lhotáková, Lucie Kubínová, Jana Albrechtová

Supplementary Table S1: Review of studies with results on a number of chloroplasts per mesophyll cell of different plant species (most studied plant species: families *Brassicaceae, Fabaceae*, *Chenopodiaceae, Poaceae* and other families; coniferous species) with focus on the method used. SD – standard deviation, SE – standard error.

**Authors**	**Plant species**	**Plant/leaf age, leaf position**	**Number of chloroplasts per mesophyll cell**	**Method of counting**
division *Magnoliophyta* - flowering plants, class *Magnoliopsida*, order *Piperales*, family *Piperaceae*				
Ahmadabadi and Bock, 2012	*Peperomia argyreia* (Hook.f.) E. Morr	palisade parenchyma<br>spongy parenchyma	2.90±1.00 (SE)<br><br>8.85±1.72 (SE)	focusing through the cell in microscope
Ahmadabadi and Bock, 2012	*Peperomia metallica* Linden and Rodigas	palisade parenchyma<br>spongy parenchyma	2.24±0.61 (SE)<br><br>6.52±2.24 (SE)	focusing through the cell in microscope
Ahmadabadi and Bock, 2012	*Peperomia peduncularis* Sodiro	palisade parenchyma<br>spongy parenchyma	2.62±0.80 (SE)<br><br>7.39±2.00 (SE)	focusing through the cell in microscope
Ahmadabadi and Bock, 2012	*Peperomia serpens* (Sw.) Loudon	palisade parenchyma<br>spongy parenchyma	16.77±3.33 (SE)<br><br>17.13±3.90 (SE)	focusing through the cell in microscope
division *Magnoliophyta* - flowering plants, class *Liliopsida* - monocotyledons				
order *Alismatales*, family *Araceae* - aroids				
Gopi et al., 2008	*Amorphophallus campanulatus* Blume ex Decne (Dennst.) Nicolson	150 days after planting, palisade parenchyma<br>spongy parenchyma	13.21<br><br>15.23	not specified, probably profile counting, thin transverse sections of the leaf, light microscope
order *Poales*, family *Poaceae*				
Hassan and Wazudin, 2000	*Oryza sativa L.*	4 months after germination	24.3	comparing photographs from three planes of focus
Warner et al. (1987)	*Panicum virgatum* L.	tetraploid	9.4±0.7 (SD)	maceration

Wardley et al., 1984	*Triticum aestivum* L. cv Egret	seedlings<br>senescence after 14 d	185±12 (SE)<br><br>140±25 (SE)	maceration
Dean and Leech, 1982	*Triticum aestivum* var. Maris Dove	seedlings, distance from leaf base:<br>2 cm<br>4 cm<br>6 cm<br>8 cm<br>10 cm	 <br><br>69±4 (SE)<br>90±5 (SE)<br>109±7 (SE)<br>133±6 (SE)<br>135±7 (SE)	maceration
Boffey et al., 1979	*Triticum aestivum,* var. Maris Dove	seedlings, different distance from leaf base	45 to about 150	maceration
Ellis and Leech, 1985	*Triticum monococcum* L.	seedlings, 7 days after sowing, first leaves, fully expanded tissue - 8.0 to 8.5 cm from the leaf base	46	maceration
Possingham, 1980 **review**	*Triticum vulgare* Vill.	leaf number 4,days after sowing:<br>11<br>17<br>25	 <br>5<br>10<br>150	maceration
division *Magnoliophyta* - flowering plants, class *Rosopsida* – eudicots				
order *Brassicales*, family *Brassicaceae* - crucifers				
Meyer et al., 2006	*Arabidopsis thaliana* L. (thale cress) wild type cv. Columbia	seedlings, 10 days after germination	203±38 (N/A)	not specified
Pyke and Leech, 1992	*Arabidopsis thaliana* L. Heynh. var Landsberg erecta	first leaves during the course of leaf expansion – between 9 and 22 d after sowing	121	maceration
Jin et al., 2011	*Arabidopsis thaliana* L. Heynh., wild-type Columbia	stage 5.10 (when bolting had just commenced)	8.5±2.2 (SD)	profile counting
Teng et al., 2006	*Arabidopsis thaliana* L. Heynh., wild-type Columbia	stage 5.0 (fully expanded	8.90±2.9 (SD)	not specified, probably profile counting, TEM thin

		rosette leaves)		sections
Stettler et al., 2009	*Arabidopsis thaliana* L., ecotype Columbia	4-weeks old plants (rosettes with 15-16 leaves), young leaves (number 6-8) mature leaves (number 13-15)	11.1±0.4 (SE) 73.1±2.0 (SE)	maceration
Marrison et al., 1999	*Arabidopsis thaliana* L., Landsberg erecta	fully expanded first leaves	120	maceration
order *Cucurbitales*, family *Cucurbitaceae*				
Zechmann et al., 2003	*Cucurbita pepo* L. subsp. *pepo* var. *styriaca* Greb.	5th leaves: palisade cell layer spongy parenchyma youngest fully developed leaves palisade cell layer spongy parenchyma	12±1 (N/A) 7±2 (N/A) 10±1 (N/A) 6±1 (N/A)	profile counting
order *Fabales*, family *Fabaceae* - legumes				
Coate et al., 2012	*Glycine dolichocarpa* Tateishi and H. Ohashi, natural allotetraploid	palisade parenchyma	19± 3 (SD)	from 3D reconstructed images (confocal microscope)
Molin *et al.*, 1982	*Medicago sativa* L.	diploid	21.4	maceration
Possingham, 1980 **review**	*Phaseolus vulgaris* L.	first pr. leaf, days after sowing: 2 8	8 50	maceration
Lamppa *et al.*, 1980	*Pisum sativum* L., cv. Alaska wilt-resistant	leaves of length: 4 mm 5.5 mm 7.0 mm	24±10 (SD) 35±11 (SD) 47±13 (SD)	maceration

		10 mm<br>11 mm<br>11.5 mm<br>12.0 mm<br>12.3 mm<br>12.5 mm	55±16 (SD)<br>64±20 (SD)<br>57±15 (SD)<br>55±16 (SD)<br>50±12 (SD)<br>44±12 (SD)	
order *Solanales*, family *Solanaceae*				
Possingham, 1980 **review**	*Nicotiana tabacum* L.	leaf number 8 days after sowing:<br>23 days<br>31 days	 <br>10-20<br>200	maceration
Ahmadabadi and Bock, 2012	*Solanum lycopersicum* Mill. cv. IPA-6	palisade parenchyma<br>spongy parenchyma	28.59±9.54 (SE)<br>19.13±6.30 (SE)	focusing through the cell in microscope
Tymms et al., 1983	*Beta vulgaris var. Fordhook giant*	leaf size:<br>2-3 cm<br>25-30 cm	 10.8±0.39 (SE)<br>65.4±4.8 (SE)	maceration
order *Caryophyllales*, family *Chenopodiaceae*				
Yamasaki *et al.*, 1996	*Chenopodium album* L.	newly expanded second leaves	about 90	maceration
Possingham and Smith, 1972	*Spinacia oleracea* L.	young, 2 cm long leaves	31	maceration
Possingham, 1980 **review**	*Spinacia oleracea* L.	leaf number 5 days after sowing:<br>20 days<br>34 days	 <br>10-50<br>200	maceration
Possingham and Saurer, 1969	*Spinacia oleracea* L. (American Round-Seeded Spinach)	21 d old plants, palisade cells, leaf position (from lowest on stem):<br>1 and 2<br>3 and 4<br>5 and 6<br>7 and 8<br>9	 <br>540<br>380<br>320<br>65<br>40	maceration
Chaly *et al.*, 1980	*Spinacia oleracea* L.		60-80	maceration
order *Asterales*, family *Asteraceae*				
Possingham,	*Helianthus annuus* L.	2nd pr. leaf - days after		maceration

1980 **review**		sowing:<br>21 days<br>50 days	 19<br>50	
order *Vitales*, family *Vitaceae*				
Yang et al., 2012	*Vitis vinifera* L.		5-9	profile counting
division *Pinophyta* – conifers, class *Pinopsida*, order *Pinales*				
family *Taxaceae*				
Maslova et al., 2009	*Taxus cuspidate* S. et Z. ex E. (yew)	60 years,<br>August<br>December<br>March<br>April	 16.0±1.1 (SE)<br>33.0±1.9 (SE)<br>29.0±1.6 (SE)<br>22.0±0.9 (SE)	profile counting
family *Cupressaceae*				
Maslova et al., 2009	*Thuja occidentalis* (arborvitae "green")	20 years,<br>August<br>December<br>March<br>April	 6.0±0.9 (SE)<br>25.0±1.0 (SE)<br>22.0±1.2 (SE)<br>21.0±1.4 (SE)	profile counting
Maslova et al., 2009	*Thuja occidentalis* f. "Reingold" (arborvitae "yellow")	20 years,<br>August<br>December<br>March<br>April	 6.0±0.3 (SE)<br>16.0±0.9 (SE)<br>26.0±1.4 (SE)<br>24.0±1.2 (SE)	profile counting

Additional references:

Ahmadabadi M., Bock R. 2012. Plastid division and morphology in the genus Peperomia. *Biologia Plantarum* **56,** 301-306.

Wardley TM, Bhalla PL, Dalling MJ. 1984. Changes in The Number and Composition of Chloroplasts during Senescence of Mesophyll-Cells of Attached and Detached Primary Leaves of Wheat (*Triticum aestivum* L). *Plant Physiology* **75**, 421-424.

Warner DA, Ku MSB, Edwards GE. 1987. Photosynthesis, Leaf Anatomy, and Cellular-Constituents in the Polyploid C-4 Grass Panicum-Virgatum. *Plant Physiology* **84**, 461-466.

Yang D, Li S, Li M., Yang X, Wang, W, Cao Z, Li W. 2012. Physiological Characteristics and Leaf Ultrastructure of a Novel Chlorophyll-deficient Mutant of *Vitis venifera* Cultured in vitro. *Journal of Plant Growth Regulation* **31,** 124-135.

See separate files:

Supplementary Video S1. The 3D stack of 16 serial optical cross sections 2 µm apart acquired by confocal microscopy using 20× objective, see Material and Methods section. The slice movie was created in the Corel PHOTO-PAINT X4 (Corel Corporation).

Supplementary Video S2. The 3D reconstruction of mesophyll arrangement created by volume rendering from images of 16 serial optical sections rotating in 3D space. The 3D volume rendered movie was captured in the Ellipse (ViDiTo, Slovakia) module Contours and processed by Corel PHOTO-PAINT X4 (Corel Corporation).

Supplementary Video S3. The 3D model of a simplified mesophyll cell with 210 chloroplasts (modelled by surfaces of oblate ellipsoids) made in IRIS Explorer (NAG, UK). The model was surface rendered in Cortona 3D viewer and the movie was recorded by Corel CAPTURE X4 (Corel Corporation).

Next Page:

Supplementary Figure S1. Norway spruce needle longitudinal median section by confocal microscopy using 20× objective. Autofluorescence of chlorophyll in chloroplasts was detected in the red channel and autofluorescence of phenolics was detected in the green channel. Asterisks – intercellular spaces, arrows mesophyll cells. Note the vertical layers of tightly connected mesophyll cells surrounded by intercellular spaces.

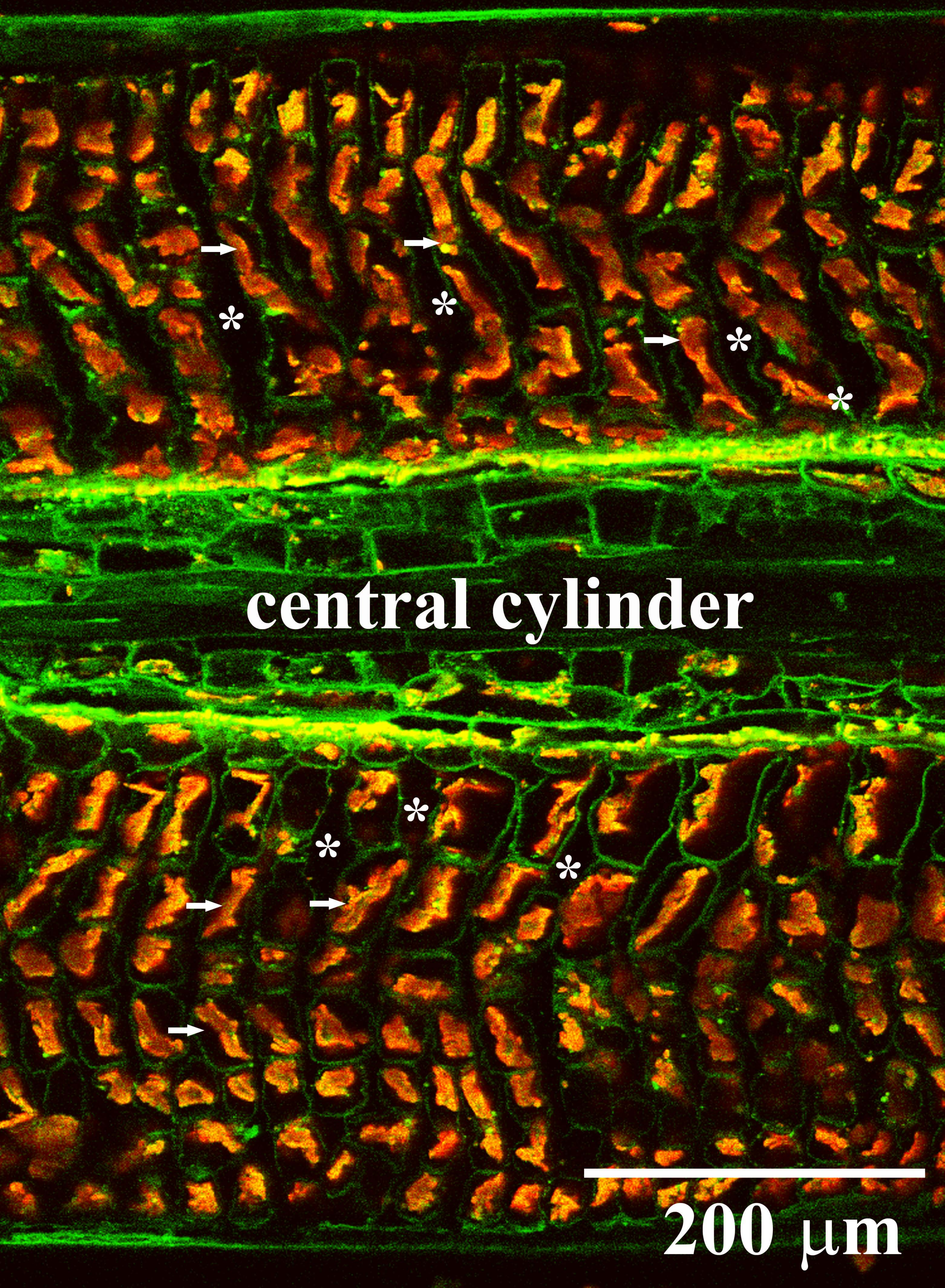
central cylinder
200 μm
